# An 8 week open-label interventional multicenter study to explore the lung clearance index as endpoint for clinical trials in cystic fibrosis patients ≥8 years of age, chronically infected with *Pseudomonas aeruginosa*

**DOI:** 10.1186/s12890-020-01201-y

**Published:** 2020-06-12

**Authors:** Sivagurunathan Sutharsan, Susanne Naehrig, Uwe Mellies, Christian Sieder, Jörg Ziegler

**Affiliations:** 1 Division for Cystic Fibrosis, Department of Pulmonary Medicine, University Medicine Essen – Ruhrlandklinik, Essen, Germany; 2 Cystic Fibrosis Center for Adults, University Hospital Munich, Med. Klinik V, Munich, Germany; 3 Pediatric Pulmonology and Sleep Medicine, Children’s Hospital, University of Duisburg-Essen, Essen, Germany; 4 Novartis Pharma GmbH, Nürnberg, Germany

**Keywords:** Cystic fibrosis, Forced expiratory volume in 1 second, Lung clearance index, Tobramycin, *Pseudomonas aeruginosa*, Antibiotics, Clinical trials

## Abstract

**Background:**

Forced expiratory volume in 1 second (FEV_1_) is the only parameter currently recognized as a surrogate endpoint in cystic fibrosis (CF) trials. However, FEV_1_ is relatively insensitive to changes in the small airways of patients with milder lung disease. This pilot study aimed to explore the lung clearance index (LCI) as a marker for use in efficacy trials with inhaled antibiotics in CF.

**Methods:**

This open-label, single-arm study enrolled CF patients with *Pseudomonas aeruginosa* infection, who were treated with tobramycin (28-day on/off regime). FEV_1_, LCI and bacterial load in sputum (CFU) were assessed at baseline, after 1, 4 and 8 weeks of treatment.

**Results:**

All patients (n = 17) showed elevated LCI of > 11 despite 3 patients having normal FEV_1_ (> 90% predicted) at baseline. Overall, LCI improved in 8 (47%) patients and FEV_1_ in 9 (53%) patients. At week 4, LCI improved by 0.88, FEV_1_ increased by 0.52%, and *P. aeruginosa* reduced by 30,481.3 CFU/mL. These changes were however statistically non-significant. Six adverse events occurred in 5/17 (29.4%) patients, most of which were mild-to-moderate in severity.

**Conclusions:**

Due to the low evaluable sample size, no specific trend was observed related to the changes between LCI, FEV_1_ and CFU. Based on the individual data from this study and from recently published literature, LCI has been shown to be a more sensitive parameter than FEV_1_ for lung function. LCI can be hypothesized to be an appropriate endpoint for efficacy trials in CF patients if the heterogeneity in lung function is limited by enrolling younger patients or patients with more milder lung disease and thus, limiting the ventilation inhomogeneities.

**Trial registration:**

The study is registered with ClinicalTrials.gov, identifier: NCT02248922.

## Background

Cystic fibrosis (CF) is a life-limiting, autosomal recessive genetic disease [1], characterized by a reduced quantity and/or function of the cystic fibrosis transmembrane conductance regulator (CFTR) protein, leading to an impaired mucociliary clearance of the airways, followed by chronic inflammation, infection and thus progressive and irreversible loss of lung function [2]. The main driver of lung function decline beyond infancy in people with CF is the chronic infection with *Pseudomonas aeruginosa* [3]. Although it is a complex multiorgan and multifactorial disease, the chronic lung infection by *P. aeruginosa* is the primary cause of morbidity. Therefore, an antibiotic maintenance therapy is the preferred standard treatment in these patients [2]. Inhaled tobramycin typically given in 28 day cycles of 1 month on and 1 month off periods, is recommended for treating individuals with CF aged ≥6 years, who have mild to moderate lung disease with persistent *P. aeruginosa* infection [4].

Currently, forced expiratory volume in 1 second (FEV_1_) and rate of FEV_1_ decline are the only accepted surrogate markers for assessing lung function decline [5]. However, with significant advances in the care of patients with CF, such as the newly introduced CFTR-targeted therapies and considerable improvement in survival rates, the rate of decline in FEV_1_ has slowed [6, 7], leading to a preservation of spirometric lung function within the normal range into young adulthood [8, 9]. Moreover, FEV_1_ is insensitive during early stages of lung disease resulting in normal spirometry findings, despite the progression of structural damage in lungs, for example in bronchiectasis. Therefore, the reliability on FEV_1_ as an indicator of disease progression especially in the early stages of lung disease has become questionable [9, 10]. Increasing evidence suggest that in patients with CF, pulmonary deterioration starts early in life and can occur in the absence of respiratory symptoms at normal lung function in terms of FEV_1_ [11]. Thus, there is a need for early intervention before onset of irreversible lung damage based on alternative clinically sensitive surrogate markers for detection of early CF lung disease [12].

A multiple breath washout (MBW) test assessing ventilation inhomogeneity has been shown to be a sensitive lung function test to detect early pathology in patients with CF [12]. The most commonly reported MBW outcome is the lung clearance index (LCI), which is a robust, noninvasive technique to detect early lung disease changes across all age groups [13, 14]. Consequently, LCI has been evaluated as a new sensitive surrogate endpoint for measuring trial outcomes focusing on the early stages of disease [13]. LCI was used for the first time as the primary study endpoint in a pivotal trial in children (6–11 years) with CF treated with an oral combination of CFTR modulators, lumacaftor (LUM) and ivacaftor (IVA). Treatment with LUM/IVA, demonstrated a statistically significant improvement in lung function, as measured by LCI_2·5_ and percent predicted FEV_I_ (ppFEV_1_), versus placebo in children with CF homozygous for F508del-CFTR [15]. However, there is still very limited data available on the use of LCI as a clinical endpoint for CF patients treated with inhaled antibiotics. Recent evidence from longitudinal studies also suggest a between-visit variability in the LCI with increasing severity of the lung disease [16].

The aim of the present study was to explore the clinical utility of LCI for assessing short-term clinical response to inhaled tobramycin antibiotic therapy in patients with CF positive for *P. aeruginosa,* aged 6 years and older with mild to moderate lung disease.

## Methods

### Study design and patients

This was an open-label, single arm study in CF patients with chronic *P. aeruginosa* infection. The study consisted of screening period (5 to 26 days) to test the presence of *P. aeruginosa* (Visit 1), a baseline visit (Visit 2), a visit 1 week after baseline visit (Visit 3), followed by a visit after 28 days on-treatment period (Visit 4) and subsequently a visit after 28 days off-treatment period (Visit 5, end of study). All patients were treated with tobramycin monotherapy, either as an inhalation solution (TIS) 300 mg twice daily (BID) or as an inhalation powder (TIP) 128 mg BID. FEV_1_, LCI and bacterial load in sputum (colony forming units [CFU]) were assessed at baseline, after 1, 4 and 8 weeks of treatment. Spirometric and LCI assessments were conducted before or at least 1 hour after the inhalation of the drug. MBW measurements to assess the LCI were performed with the ExhalyzerD (Eco Physics GmbH, Munich, Germany). The study design was approved by the independent ethics committee and institutional review boards; and was in accordance with the Declaration of Helsinki.

The study enrolled patients from seven sites in Germany, diagnosed with CF aged 6 to 50 years, with a chronic infection of airways with *P. aeruginosa* within the last 12 months and at screening. Patients with an elevated LCI of ≥7.5 [17] and FEV_1_ of ≥50% at screening, and those receiving inhaled tobramycin monotherapy in 28 days on/off regime in the past 3 month before screening were included. Informed consent was obtained from patients for having their data collected. Patients receiving more than one class of inhaled antipseudomonal antibiotic during the study or in the past 56 days prior to the baseline visit, or who used oral or intravenous antipseudomonal antibiotics within 28 days prior to the on-phase of the study drug or loop diuretics within 7 days prior to the first study medication administration, were excluded.

### Study assessments

The primary endpoint was change in LCI after 4 weeks of treatment versus baseline. Secondary endpoints included change in FEV_1_ and CFU after week 4 following treatment; change of LCI after week 1 following treatment; change of LCI, FEV_1_ and CFU between week 4 (end of the study drug inhalation in the current treatment cycle) and week 8 (prior to the start of the study drug inhalation in the following treatment cycle); and correlation between the changes of LCI, FEV_1_ and CFU after weeks 1, 4 and 8 versus baseline, respectively.

For LCI assessment, the multiple breath washout device Exhalyzer D (Ecomedics, Zurich, Switzerland) was used, with Nitrogen as the tracer gas. All equipment and the device software were identical throughout the sites. LCI was determined according to the Exhalyzer D MBW SOP of the manufacturer that is based on the current MBW guidelines of the European Cystic Fibrosis Society (ECFS), and all study sites were provided with a device specific training before start of the study.

The mean of two acceptable MBW measurements was used for analysis if the coefficient of variation (CV) for LCI was ≤5%, else another MBW measurement was performed (functional residual capacity [FRC] not > 25%). The mean of three acceptable MBW measurements was used, if the CV for LCI was ≤10% (FRC not > 25%). One more MBW measurement was to be performed otherwise. MBW was repeated four times, and the mean of two measurements with the smallest difference in LCI was used. Single MBW assessments were stopped immediately if more than one cough event occurred during the wash-out phase, if there was a continuous decrease of the carbon dioxide concentration for > 60 s, and if there was a change in the breathing pattern of more than 20%. For the measurements used to calculate the mean value, a quality control was done by a central assessor (MBW specialist of the manufacturer).

### Statistical analysis

Assuming a change of 5% in FEV_1_ with an intraindividual standard deviation of 9% between the on- and off-treatment cycles, 28 patients would provide 80% power on a 2-sided 5% significance level to detect this change. To compensate for protocol deviations and dropouts, 35 patients were planned to be recruited into this trial. All variables were summarized using descriptive statistics. Analyses were performed by using SAS Version 9.4.

Changes in LCI from baseline were analyzed using an analysis of variance (ANOVA) model. Estimates over time were presented as least squares (LS)-means, pairwise LS-mean differences along with 95% confidence intervals (CI) and 2-sided p-values for the pairwise differences between visits. If the baseline value was missing, then change was calculated from the data recorded at the screening. The primary analysis included patients with a valid observation at the baseline and post-baseline visits.

## Results

**Fig. 1:**
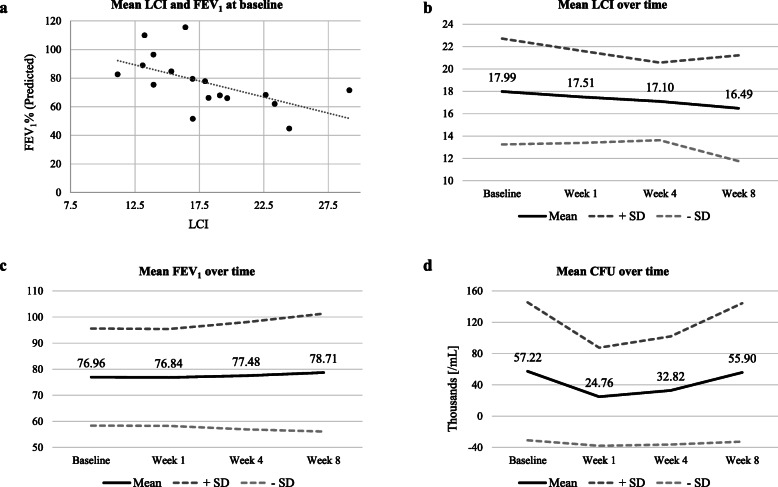
Lung function parameters (LCI and FEV_1_) and CFU at baseline and over time CFU, colony forming units; FEV_1_, forced expiratory volume in 1 second; LCI, lung clearance index; SD, standard deviation

In total, 17 patients (5 patients treated with TIS and 12 patients with TIP) were enrolled and completed the study. The majority of the patients (14 patients, 82.4%) were aged > 17 years, with an overall mean age of 26.4 ± 9.99 years and body mass index (BMI) of 21.1 ± 2.46 kg/m^2^. The study had a higher proportion of men (64.7%) versus women (35.3%). All patients provided valid MBW measurements with three trials each, at each visit, and had complete data on LCI and FEV_1_. Not all patients were able to expectorate sputum and thus, complete CFU data were only available for 10 patients. At baseline, mean LCI was 17.99 ± 4.73 (95% CI: 11.16–29.14) and FEV_1_ was 76.96 ± 18.60% predicted (95% CI: 44.66–115.5) (Fig. 1a). All patients demonstrated considerably elevated LCI of > 11 (normal range 6.5–7.5) despite 3 patients having a normal lung function (FEV_1_ > 90% predicted) at baseline. The demographics and characteristics of patients at the baseline are presented in **Table****1**.

**Table 1:** Patient demographics and baseline characteristics (*N* = 17)

Variable	
Age, years	26.4 (9.99)
Men, n (%)	11 (64.7)
Height, cm	171.12 (12.8)
Weight, kg	62.6 (12.9)
Waist circumference, cm	85.1 (22.6)
BMI, kg/m^2^	21.1 (2.5)
LCI	17.99 (4.7)
FEV_1 _(%)	76.96 (18.6)

Data are expressed as mean (SD) unless indicated otherwise

*BMI* body mass index, *FEV*_*1*_ forced expiratory volume in 1 second, *LCI* lung clearance index, *SD* standard deviation

The mean LCI improved slightly over time from baseline. At week 4, LCI improved by 0.88 (17.10 ± 0.84; 95% CI: 15.31–18.88) from baseline, which was statistically non-significant (*p* = 0.41). Overall, 8 (47%) patients showed an improved LCI at week 4 from baseline. At least 3 patients showed worsening of LCI despite improvement in FEV_1_ (Table 2).

**Table 2:** Lung function parameters after visit 4 versus baseline

Patient #	Age	FEV_**1**_ at baseline (% predicted)	dLCI in % of baseline	dFEV_**1**_ in % of baseline	dCFU [/g]
1	17	82.6	29.8%	11.0%	−17,120
2	17	84.7	3.8%	10.6%	–
3	37	71.5	−42.4%	7.2%	−224,700
4	19	68.2	−24.6%	7.0%	–
5	19	115.5	−19.8%	4.4%	–
6	24	109.9	10,5%	2.7%	−12,200
7	35	77.8	−3.0%	2.1%	−12,640
8	37	79,5	6.7%	0.9%	−40
9	22	44.7	−24.1%	0.6%	20,800
10	18	65.9	−4.8%	0.0%	− 225,600
11	41	61.9	−4.6%	0.0%	− 8800
12	30	51.5	−15.6%	−1.0%	100
13	8	88.9	− 6.9%	−2.2%	732
14	43	101.1%	8.7%	−5.1%	14,000
15	34	67.9%	17.1%	−6.9%	−10,040
16	23	75.3%	4.8%	−12.5%	29,600
17	24	68.6%	36.2%	−13.0%	–

*CFU* colony forming units, *d* difference at week 4 versus baseline, *FEV*_*1*_ forced expiratory volume in one second, *LCI* lung clearance index

The mean FEV_1_ increased by 0.52% (77.48 ± 20.56; 95% CI: 66.91–88.05) at week 4 from baseline, which was not statistically significant (p = 0.70). At week 4, 9 patients (53%) showed an improved FEV_1_ and *P. aeruginosa* reduced by 30,481.3 CFU/mL (26,113.0 CFU/mL; 95% CI: (− 32,035.02–84,261.05) as compared to baseline (56,594.3). However, the change in CFU was not statistically significant (p = 0.35). The mean LCI improved slightly (− 0.479) after 1 week of drug inhalation (*p* = 0.59). The changes in LCI (*p* = 0.42), FEV_I_ (*p* = 0.39), and CFU (p = 0.35) from week 4 to week 8 were statistically non-significant (Fig. 1b-d). A statistically significant positive correlation was observed between LCI and CFU at week 1 (*r* = 0.59, *p* = 0.0321) and week 4 (r = 0.79, p = 0.0046) from baseline. A negative correlation (*r* = − 0.64) was seen between FEV_1_ and CFU at week 4 from baseline, which was statistically significant (p = 0.0462).

Overall, 6 adverse events (AEs) occurred in 5/17 patients (29.4%) (3 patients on TIP and 2 patients on TIS), most of which were related to respiratory, thoracic and mediastinal disorders (*n* = 3, 17.6%). Most of these were mild or moderate in severity (Table 3).

**Table 3:** Adverse event

System organ class	TIS (***N*** = 5), ***n*** (%)	TIP (N = 12), ***n*** (%)	Overall (***N*** = 17), ***n*** (%)
Any MedDRA system organ class	2 (40.0)	3 (25.0)	5 (29.4)
Injury, poisoning and procedural complications	1 (20.0)	0 (0.0)	1 (5.9)
Sunburn	1 (20.0)	0 (0.0)	1 (5.9)
Investigations	1 (8.3)	0 (0.0)	1 (5.9)
Forced expiratory volume decreased	1 (8.3)	0 (0.0)	1 (5.9)
Respiratory, thoracic and mediastinal disorders	2 (16.7)	1 (20.0)	3 (17.6)
Cough	1 (8.3)	0 (0.0)	1 (5.9)
Haemoptysis	0 (0.0)	1 (20.0)	1 (5.9)
Obstructive airways disorder	1 (8.3)	0 (0.0)	1 (5.9)

*TIS* Tobramycin inhalation solution, *TIP* Tobramycin inhalation powder. Percentages are based on N of each group in safety set. A patient with multiple occurrences of an AE is counted only once in the AE category. A patient with multiple adverse events within a primary system organ class is counted only once in each system organ class total row. MedDRA version 20.0 has been used for coding of adverse events

## Discussion

**Fig. 2:**
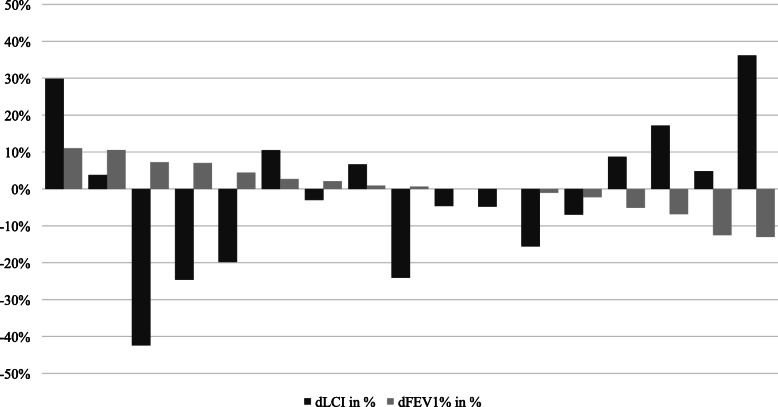
Matched pairs of relative changes in LCI and FEV_1_ at week 4 from baseline d, difference; FEV_1_, forced expiratory volume in 1 second; LCI, lung clearance index

It has been shown that lung damage induced by inflammation precedes FEV_1_ decline [10]. LCI derived from MBW reflects ventilation defects in the respiratory tract, including the early dysfunction in small airways, which are not easily identified with traditional spirometry methods [13]. Therefore, the present study assessed the clinical utility of LCI for assessing the response to inhaled tobramycin antibiotic therapy (both nebulized and powder inhalers) in patients with CF positive for *P. aeruginosa*. The use of both nebulized and powder inhalers in this study was based on evidence from previous non-inferiority studies suggesting similar efficacy between the two aerosol therapies [18]. In the present study, three patients had a significantly elevated LCI despite having a normal FEV_1_% at baseline. In addition, relative changes in LCI at week 4 versus baseline for individual patients were higher than the respective FEV_1_ differences (Table 2 and Fig. 2), indicating a higher sensitivity of lung function assessment with MBW compared to classical spirometry. Similar findings from previously published data also showed that LCI provides a more sensitive overall estimate of ventilation inhomogeneity compared to FEV_1_, at least in patients with mild lung disease [19].

There is increasing evidence on the clinical utility of LCI. In addition to LCI being used as a clinical endpoint [5, 15], it has also been used to assess mucociliary clearance in young children [20] treated with CFTR-modulators [15] and to monitor disease progression [21], or both [22]. However, this study with inhaled tobramycin demonstrated that changes in LCI, FEV_1_ and CFU at week 1, 4 and 8 were not statistically significant. There was no specific trend observed with respect to correlation between the changes of LCI, FEV_1_ and CFU after week 1, 4 and 8 versus baseline. This may be attributed to the heterogeneity in LCI response to antibiotic treatment seen in the individual data for MBW and spirometry shown in Fig. 2. These results are in line with previously published studies [23, 24] and pooled analyses [25]. Despite the use of a more efficient therapy (inhaled formulation) in terms of an increased bioavailability of tobramycin in sputum we observed heterogeneity in the present study which is comparable to the findings seen with intravenous antibiotic treatment in children with CF [23]. In contrast to the outcome from the present study, a short-term study (1 month) with 32 CF children on intravenous antibiotic treatment showed a significant improvement in LCI compared with FEV_1_ [26]. However, data about LCI as outcome measure for short-term efficacy of antibiotics in CF remains very limited.

The observed heterogeneity in lung function response to therapy can partly be attributed to the non-responders, which could be due to a few patients becoming refractory to inhaled antibiotics [27]. Further, there were single cases of negative, or positive change in CFU in some patients however, such findings are not conclusive, also because not all patients were able to expectorate sputum and thus, CFU data was missing for some patients. In addition, CFU assessment in general is characterized by huge intraindividual variation and therefore, any non-significant findings have to be evaluated with caution. Based on this data, one may speculate that therapy response in these patients with mild to moderate lung disease, chronically treated with inhaled tobramycin was weak and/or heterogeneous. At least 3 patients showed worsening of LCI despite an improvement in FEV_1_, which could be due to increased ventilation inhomogeneity in newly ventilated lung regions following inhaled antibiotic therapy (eg. by reduced air-trapping) [24, 28]. However, it is important to note that the small evaluable sample size of the present study makes it challenging to draw clear conclusions.

In addition, chest physiotherapy to remove sticky mucus from the airways, prior to lung function testing may also lead to heterogeneity in LCI response. The inherent ventilation inhomogeneity combined with the clearance of mucus plugged airways (after chest physiotherapy) which were previously poorly ventilated (at baseline) leads to an increased LCI [29]. Indeed, a mean reduction of 0.2 in LCI response 30 min after physiotherapy was observed in CF patients with lung disease of varying severity, suggesting unpredictability of short-term physiotherapy on LCI responses [30]. Similar to previously reported studies, the standard physiotherapy performed in the present study is strongly dependent on the therapist’s discretion as well as on the daily performance and adherence of the individual CF patient. Although chest physiotherapy is typically performed before inhalation and is done at least 1 h prior to MBW, there is no guidance on the timing for the physiotherapy at each visit. Hence, chest physiotherapy could have affected the outcome of lung function testing in this study to a certain degree. A detailed documentation of the timing and type of physiotherapy relative to MBW lung function testing in future clinical trials using LCI as a study endpoint is therefore recommended [29].

Based on the results from the present study as well as from available literature, it can be hypothesized that LCI can be an appropriate endpoint for efficacy trials in CF patients if the heterogeneity in lung function is limited by enrolling younger patients or patients with more milder lung disease and thus, limited ventilation inhomogeneities. Furthermore, including younger patients with milder lung disease and specifically defined time points for chest physiotherapy; while excluding false negative changes in lung function outcomes resulting from physiotherapy is recommended.

During the study period six AEs were reported, all of which were mild and moderate in severity, which is in line with previously published phase III data [31, 32] and the clinical experience with inhaled tobramycin.

A major limitation of the present study is the small sample size because of difficulties with patient enrollment and recruitment. The standard of care changed during the setup of the study, i.e., the majority of *P. aeruginosa* positive CF patients were to be treated with continuous inhaled sequential antibiotic combinations [33]. Hence, enrollment of patients on monotherapy was a challenge and thus, the study could include only 17 patients versus 35 patients that were planned to be enrolled. This was the primary reason for the premature termination of the study. Furthermore, it is important to consider the differences in the reference values of upper limit of normal of LCI while enrolling patients belonging to different age groups, for accurate interpretation of results.

## Conclusions

In summary, the changes in LCI, FEV_1_ and CFU were statistically non-significant at week 1, 4 and 8 when compared with baseline which may be due to the heterogeneity in LCI response to antibiotic treatment and the low evaluable sample size.

Even if LCI is not identified as the ideal clinical efficacy endpoint for studies with antibiotic treatment (either intravenous or inhaled), it does not jeopardize the overall value of LCI as a more sensitive lung function parameter for CF patients with mild to moderate lung disease. Considering previously published data, LCI seems to be a suitable endpoint for drugs affecting the mucociliary clearance and to assess disease progression in cystic fibrosis. However, LCI alone does not seem to be the ideal clinical endpoint for efficacy studies with antibiotic treatment (either intravenous or inhaled) in small groups of CF patients.

## Supplementary information

**Additional file 1: Supplementary Table 1.** List of Independent Ethics Committees or Institutional Review Boards.

## Data Availability

The data generated during and/or analyzed during the current study are available from the corresponding author upon reasonable request.

## Abbreviations

AE: Adverse events
ANOVA: Analysis of variance
BID: Twice daily
BMI: Body mass index
CF: Cystic fibrosis
CFTR: Cystic fibrosis transmembrane conductance regulator
CFU: Colony forming unit
CI: Confidence intervals
d: Difference
FEV_1_: Forced expiratory volume in one second
IVA: Ivacaftor
LCI: Lung clearance index
LS: Least squares
LUM: Lumacaftor
MBW: Multiple breath washout
*P. aeruginosa*: *Pseudomonas aeruginosa*
ppFEV_1_: Percent predicted FEV_I_
SD: Standard deviation
TIP: Tobramycin inhalation powder
TIS: Tobramycin inhalation solution
