# Supplementary material for: An 8 week open-label interventional multicenter study to explore the lung clearance index as endpoint for clinical trials in cystic fibrosis patients ≥8 years of age, chronically infected with Pseudomonas aeruginosa
Source: BMC Pulm Med. 2020 Jun 12;20:167. doi: 10.1186/s12890-020-01201-y (PMC7291662; doi:10.1186/s12890-020-01201-y)
Supplement: Supplementary file 1 — Additional file 1: Supplementary Table 1. List of Independent Ethics Committees or Institutional Review Boards. [file 12890_2020_1201_MOESM1_ESM.docx]

**Supplementary table 1.** List of Independent Ethics Committees or Institutional Review Boards

| **Ethics Committees or Institutional Review Board** | **Address** |
| --- | --- |
| 1. Universitätsklinikum Essen Medizinische Fakultät  der Universität Duisburg-Essen Ethik-Kommission | Robert-Koch-Straße 9-11 45147 Essen |
| 1. Ethik Kommission der Medizinischen Fakultät der  Ruhr-Universität Bochum | Gesundheitscampus 33 44801 Bochum |
| 1. Universitätsklinikum Jena   Ethik-Kommission | Bachstr. 18 07740 Jena |
| 1. Landesamt für Gesundheit und Soziales  Ethik-Kommission des Landes Berlin | Fehrbelliner Platz 1 10707 Berlin |
| 1. Friedrich-Alexander- Universität Erlangen- Nürnberg Medizinische Fakultät Ethik-Kommission | Krankenhausstr. 12 91054 Erlangen |
| 1. Johann Wolfgang Goethe- Universität Universitätsklinikum Ethik-Kommission  des Fachbereichs Medizin | Theodor-Stern-Kai 7 60590 Frankfurt am Main |
| 1. Ludwig-Maximilians- Universität München Klinikum  der Universität Ethik- Kommission | Pettenkoferstr. 8a 80336 München |
| 1. Technische Universität Dresden Medizinische Fakultät Carl Gustav Carus Ethik-Kommission | Fetscherstr. 74 01307 Dresden |
| 1. Universitätsklinikum Essen Medizinische Fakultät  der Universität Duisburg-Essen Ethik-Kommission | Robert-Koch-Straße 9-11 45147 Essen |
